# Energy for Conventional Water Supply and Wastewater Treatment in Urban China: A Review

**DOI:** 10.1002/gch2.201600016

**Published:** 2017-07-13

**Authors:** Kate Smith, Shuming Liu

**Affiliations:** ^1^ School of Environment Tsinghua University Haidian District Beijing 100084 China

**Keywords:** distribution, electricity, groundwater, primary treatment, secondary treatment, surface water

## Abstract

This review seeks to provide a better understanding of energy used in the urban water system in China. Electricity is a major contributor to the environmental impact of water supply and wastewater treatment, particularly in countries like China where electricity is largely generated using coal and has a significant impact on greenhouse gas emissions. Electricity use can also constitute one of the main costs for water and wastewater companies. China is an important country for the study of energy for water, particularly in urban areas where population is rapidly increasing. China's daily wastewater treatment capacity has increased dramatically over the last decade and a half, and energy use for both wastewater treatment and potable water supply has grown significantly. This paper deals with the challenge of energy for water in China. It reviews the growing body of work on energy for conventional water supply and wastewater treatment in urban China. The review covers energy for all parts of conventional water supply and wastewater treatment, including energy for sourcing, treating and distributing groundwater and surface water, and energy for primary and secondary treatment and sludge treatment and disposal.

## 1 Introduction

The water‐energy nexus is the study of the connection between water and energy. On the one hand, this connection means that water is used to generate energy. For example, for every 1000 kWh of electricity generated in the Shanghai area, 60 m^3^ of water is consumed.^1^ On the other hand, it also means that energy is used during many stages of supplying water and dealing with wastewater. To complete the cycle, energy can also be produced during the process of wastewater treatment.

When compared with the water withdrawals for energy production as a percentage of a nation's total water withdrawals, energy required during the water cycle is a small percentage of a nation's total energy requirements. For example, water used in thermoelectric power generation in the United States accounted for nearly 49% of total freshwater withdrawals,^2^ whereas estimates for electricity use in the water sector as a percentage of total electricity use in developed countries are generally under 5%. But within water supply and wastewater treatment, energy is an important component of both environmental impact and cost. The main contributor to the overall environmental burden and global warming potential of potable water production is coal‐generated electricity, which is most of the electricity produced in China.^3^, ^4^ Energy is a major contributor to the cost of producing water and treating wastewater; it can represent over 30% of annual operations and maintenance expenses in a typical water treatment plant,^5^ and between 25 and 45% of operations and maintenance expenses in wastewater treatment plants.^6^, ^7^

China is an important country for the study of energy for water, particularly in urban areas where population is rapidly increasing. China's daily wastewater treatment capacity approximately doubled between 2007 and 2013^8^ and energy use for wastewater treatment almost doubled between 2008 and 2013.^9^ Electricity consumption for potable water supply in China has more than doubled since 2000.^10^ Research on energy for water in China has increased over the past ten years. The main objective of this paper is to review the current status of energy for conventional water supply and wastewater treatment in China using the growing body of literature available.

### 1.1 Review Scope

**Figure 1:**
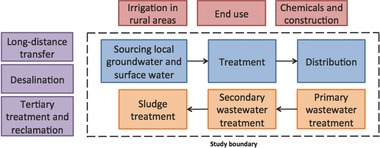
Processes shown within the dotted line are included in this review.

The scope of this review can be defined as follows. The review is restricted to electricity use for water supply and wastewater treatment in urban areas of China. (Notably, energy for (1) irrigation in rural areas, (2) energy for chemical manufacturing and plant construction and (3) residential end use (e.g. showering) are outside the scope of this review.) The majority of water sourced and treated for drinking water purposes in China is from conventional sources (i.e. groundwater and surface water sourced and used within the same province) and the majority of wastewater is treated using primary and secondary treatment. This review thus focuses on energy for conventional water supply and wastewater treatment. Alternative water sources (e.g. desalination, transfer and recycled water) and tertiary wastewater treatment are outside the scope of the review. See **Figure** **1** for the study scope.

## 2 Energy for Water Supply

An average of 0.29 kWh was required to supply one cubic metre of water to urban areas of China in 2011.^11^ This equates to 33.2 kWh for each person using water supplied through the country's central water distribution systems.^11^ A total of 1.04 × 10^10^ kWh for the supply of water to urban areas was used, which was equivalent to 0.22% of China's total electricity consumption.^11^ These indicators are shown alongside wastewater indicators in **Table 1**.

**Table 1:** Main national indicators for energy for urban water supply and wastewater treatment in China

Indicator	Value
Energy intensity of water supplya)	0.29 kWh m^–3^
Energy per capita for water supplya)	33.2 kWh cap^–1^
Total energy for urban water supplya)	1.04 × 10^10^ kWh
Energy for water supply as a percentage of national electricity consumptiona)	0.22%
Energy intensity of wastewaterb)	0.254 kWh m^–3^
Energy for wastewater as a percentage of national electricity consumptionc)	0.25%

^a)^ Smith et al.^11^

^b)^ Xie and Wang^12^

^c)^ Wang et al.^13^

### 2.1 Energy for Sourcing Water

**Figure 2:**
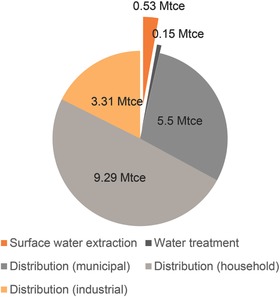
Breakdown on energy for different parts of the water supply process in Changzhou.^16^

The majority of water withdrawn in China is surface water,^14^ with use of groundwater being more common in northern provinces. Local surface water abstraction often requires less electricity than groundwater abstraction because water does not need to be lifted up to ground level. Energy use for sourcing surface water depends greatly on the distance water must be carried from the source and the topography of the area over which it is carried (i.e. elevation changes).^15^ For example, Changzhou is a large prefecture‐level city near Nanjing in southern China that relies greatly on locally sourced surface water. The city's waterworks extracted 293.75 million tons of surface water in 2009, which required 0.53 million tons coal equivalent (Mtce).^16^ As shown in **Figure** **2**, total energy used within the local waterworks was 0.15 Mtce, and total energy for distribution was 18.1 Mtce (5.5 Mtce for public use, 9.29 Mtce for distribution to households and 3.31 Mtce for distribution to industry).^16^ Thus, sourcing surface water constituted only 3% of total energy used by the city's water works for water supply.

Reliance on groundwater for municipal water supply in China depends on the location of the city. For example, in Changzhou, which is located near the Yangtze River and Tai He Lake, only 0.4% of water extracted and used in the urban water system was groundwater in 2009.^16^ In comparison, groundwater accounted for 57% of water used in Beijing and 43% of water used in Qingdao in 2011, both of which are northern cities.^17^ Extraction of groundwater primarily requires energy for pumping.^15^ The amount of energy required to raise groundwater within wells is related to (1) the pumping lift (the height separating the water level, including water drawdown caused by the well, and the base of the pump), (2) the pressure at which water is discharged from the pump, and (3) frictional resistance within the well. China's Urban Water Supply Yearbooks do not differentiate the source of water (i.e. groundwater or surface water) when providing data on energy use, so energy use for groundwater extraction by municipal water companies is difficult to obtain. For reference, groundwater extraction purely for irrigation of agriculture within urban areas is estimated to require 0.4 kWh m^–3^ in Qingdao,^18^ 0.44 kWh m^–3^ in Beijing, 0.66 kWh m^–3^ in Tianjin, with the difference between Beijing and Tianjin related to the greater average pump lift required in Tianjin (58 m) compared to Beijing (39 m).^19^ However, it should be noted that this water is often extracted from tube wells that are outside the public water supply system.

### 2.2 Energy for Treating Water

Drinking water treatment plants that treat raw surface water and groundwater in China tend to use similar treatment methods.^10^ Much of the energy used by conventional water treatment plants in China is for pumping water into the plant (sourcing) and for adding pressure to the water before it leaves the plant (distribution). Only a small amount of energy is generally used for treatment within the plant. For example, Changzhou has a population of around 3.6 million, 100% penetration of water supply services and is close to major water sources.^16^ Energy consumed within the city's waterworks for treatment of raw surface water was only 0.8% of all energy used for water supply in 2009 (which includes sourcing, treating and distributing to users).^16^ In Qingdao, northern China, local water companies estimate groundwater and surface water have similar electricity requirements of 0.376 kWh m^–3^ once the water arrives at the plant,^18^ which may include both treatment within the plant and pumping as the water leaves the plant.

Standard treatment for surface water is a combination of coagulation (and sedimentation), filtration and disinfection.^10^ Larger materials are removed through screening or through sedimentation. Energy is consumed during mixing of the raw water with the coagulant, the quantity of which will vary based on influent water quality.^20^ Sedimentation is necessarily a low‐energy process because the process relies mainly on gravity.^15^ In some cases, energy is used to run agitators in the tank and maintain a very low velocity for accelerated settling.^15^ Particles that remain in the water after coagulation and sedimentation are largely removed during filtration. Another much less common form of standard treatment for surface water in China is flotation and sedimentation, followed by filtration and disinfection.^10^ Dissolved air flotation can be used to aid flocculation and help remove dissolved gases if these cannot be sufficiently removed using coagulation and sedimentation.^15^ Energy is used to release air into the water.

Sludge produced during drinking water treatment previously constituted 74% of national sludge discharge in 2000, but this declined to around 21% in 2013,^10^ which can be linked to the rapid increase in wastewater treated in China over this time. This sludge is mostly landfilled,^10^ so energy use for sludge treatment and disposal is likely to be similar to that for wastewater treatment plants (as discussed in Section 3.3).

For groundwater, aeration and sedimentation followed by filtration and disinfection is standard treatment in China.^10^ Aeration is used to remove dissolved gases while filtration removes iron or manganese that can be present in groundwater.^15^ Chlorine is the most widely used disinfectant for drinking water in China.^21^ Based on studies conducted in other countries, chlorination of groundwater has similar energy requirements to chlorination of surface water.^15^ In the case of brackish water, standard treatment is membrane separation, followed by disinfection.^10^ When membrane processes are used (e.g. microfiltration, ultrafiltration), energy varies with the salinity of the source water, with a certain minimum energy required to pressurise water.^15^

Specific data for each treatment process in China is not available but data for the United States shows energy use for conventional drinking water treatment processes tends to be highest for in‐plant pumping (<0.06 kWh m^–3^), rapid mixing for chemical dispersion (<0.03 kWh m^–3^), coagulant feeding and flocculation (<0.02 kWh m^–3^) and gravity filtration (< 0.02 kWh m^–3^), with sedimentation, backwash pumping and chlorine feeding being the least energy intensive process (<0.01 kWh m^–3^).^15^

### 2.3 Energy for Water Distribution

Distributing water within central water distribution systems can constitute a significant percentage of energy used in the process of conventional water supply. As with sourcing water, energy for distribution is largely dependent on the distance across which water must be transferred and elevation changes between the plant and the user. For example, a new water supply system was proposed to service 560 000 people in Ningbo, southern China, in 2009.^20^ Of four proposed locations for the system's water treatment plant, the site with the largest annual electricity consumption was the only site that could not take advantage of gravity during distribution.^20^ The use of gravity reduced the carbon footprint (tons carbon dioxide equivalent per year) due to electricity use for water distribution by 27–30 times.^20^

Changzhou is an example of a city that relies on pumping to transfer water from the water treatment plant to users. In this city, distribution of treated surface water used 96% of total energy consumed by the city's conventional waterworks in 2009. Thirty percent of energy for distribution was used to pump 83.42 million tons of water for public use (i.e. landscaping and roads). Fifty‐one percent of energy for distribution was used to pump 124.63 million tons to households and 18% was used to distribute 50.24 million tons of treated water for use in industry (the majority of water used by industry was untreated water directly extracted from the source).^16^ In Beijing, distribution of water by the city's 15 water treatment plants had an average per cubic metre energy requirement of 0.19 kWh m^–3^ for years 2010 to 2012.^25^ Taipei has seven pumping stations that provide energy to distribute water in the Taipei area and energy intensity for five of these stations ranged between 0.10 and 0.26 kWh m^–3^.^22^

In China mainland, regulation suggests water distributed within central water distribution systems should have no less than 28 m of head.^23^ For this reason, all buildings of seven storeys or over need to install a pumping system to lift water to upper floors. Three studies have quantified energy use for pumping in buildings for China mainland, Taiwan and Hong Kong. Ref.^24^ focused on the roof tank system in Hong Kong residential buildings. In this system, water from the city's water distribution system is stored in a break tank near ground level, and then pumped up to rooftop tanks for distribution to every floor of the building. Ref.^24^ collected data from 22 buildings between 15 and 40 storeys high in which water was taken from the city mains and stored in a break tank before being pumped directly to a rooftop gravity tank for distribution to every floor of the building. A constant speed pump can be chosen for this system because the flow up to the roof tank is independent of the varying flow required by residents. Extrapolating the model's results to the whole of Hong Kong revealed that annual energy savings of 160 terrajoules (TJ) could be possible if high‐rise water supply systems with roof tanks were modified to include an intermediate break tank on the middle storey and that 410 TJ could be saved if tanks were included on each floor. To calculate the energy used for pumping in a standard 6‐storey building in Taiwan, Cheng^22^ used a theoretical equation for pumping power and a theoretical relationship between the energy used by a pump and the height to which water must be lifted. It was assumed the building used a roof tank system that was positioned 3–7 m above roof level. Using this method, Cheng ^22^ estimated that pumping in a 6‐storey building would require 0.14 kWh of electricity when pumping 1 m^3^ of water.

A third system used in China is the entirely pressurized booster system. This system eliminates the break tank between the central water distribution system and the first booster pump. Instead, water from the city mains enters a small, pressurized tank and is then pumped directly to the consumer using a variable speed pump. Smith, et al.^25^ compared the booster pump and break tank system and the entirely pressurized booster system. In the first system, water from the central water distribution enters a break tank at basement level or ground level and is then pumped to each floor of the building using a variable speed pump. The second system eliminates the break tank between the central water distribution system and the first booster pump. Instead, water from the city mains enters a small, pressurized tank and is then pumped directly to the consumer using a variable speed pump. The use of the pressurized tank makes pumping more efficient. The second system uses an average of 0.010 kWh m^–3^·m, compared to 0.019 kWh m^–3^·m for the first system. This means an average reduction in energy use of 45% would be possible through changing pumping systems. For a 20‐storey building in Beijing using the break tank and booster pumping system, 0.11 kWh m^–3^ is used to source and treat water, 0.19 kWh m^–3^ is used to distribute water in the central water distribution system and 1.3 kWh m^–3^ is used to pump water in the building, meaning that over 80% of all energy required to source, treat and distribute water to a 20‐storey building is used to pump water within the building.^25^ Smith, Liu, Liu, Liu and Wu^25^ extrapolated these results to a megacity in China to show that over one third of energy for water supply is associated with around one tenth of a city's population and that electricity saved by replacing 25% of break tank systems with pressurised booster systems is equivalent to around 5% of the total electricity requirements for water supply within the case city.

From the above discussion it is clear that distribution can form a major part of energy for water supply, particularly in cases where gravity distribution is not possible and local surface water is the main water source. Energy for distribution increases greatly when water users are located on the seventh floor or above. Reducing energy required to distribute treated water to consumers should be a major focus of attempts to reduce net energy for conventional water supply in China.

## 3 Energy for Wastewater

By 2013, China had built just over 3500 wastewater treatment plants, with a total capacity of 1.48 × 10^8^ m^3^ d^–1^.^26^ In 2006, the average energy consumption of 559 secondary WWTPs is 0.290kWh m^–3.^ 27 In 2009, electricity consumption per cubic metre for wastewater treated in China was 0.254 kWh m^–3^.^12^ Wastewater treatment currently accounts for about 0.25% of total electricity consumption in China.^13^

### 3.1 Primary Treatment

Of the total energy consumed within a wastewater treatment plant in China, around 25% can be attributed to the pretreatment stage, where energy use is mainly due to pumping against gravity at the inlet of the plant.^26^ In some cases, the use of air in the grit chamber during pre‐treatment can also increase energy use for pretreatment. One of Beijing's main wastewater treatment plants, Gaobeidian, is an example. In this plant, the inclusion of influent pumping and the use of an aerated grit chamber means that pretreatment is a significant energy user within the plant (32%).^28^ Primary sedimentation requires much less energy (6%).^28^

### 3.2 Secondary Treatment

After water is pumped into a wastewater treatment plant and undergoes pretreatment and primary treatment, it then passes to secondary treatment. The main technologies used in wastewater treatment plants in China are aerobic‐anoxic‐oxic (AAO), oxidation ditch, traditional activated sludge, sequencing batch reactor (SBR) and anoxic‐oxic (AO).^26^ In 2013, AAO was used in 31% of treatment plants to treat 21% of China's wastewater.^26^ Oxidation ditch technology was used in 21% of plants to treat 25% of wastewater and traditional activated sludge is used in 11% of plants to treat 15% of total wastewater treated.^26^ Processes used to a much lesser degree include membrane bioreactor, A/B process, biofilm process, biological aerated filter, biological contact oxidation process and constructed wetland.^29^

The common secondary treatment processes used in China are biological and involve significant aeration, which leads to large energy demand. Biological treatment stage can be responsible for 60–70% of energy used within a plant in China.^26^ Beijing's Gaobeidian treatment plant is a standard example, where 58% of energy is used in anaerobic‐anoxic‐oxic secondary treatment.^28^ Within the secondary treatment process itself, the aerator, wastewater and sludge pumps, and the decanters used to separate solids from effluent are the main energy consumers.^27^

In the conventional activated sludge process, wastewater (a mix of both liquid and solids) is fed into a tank. Activated sludge (a population of live microorganisms) is fed into the tank, which provides a population of live organisms that feed on organic matter and ammonia nitrogen in the wastewater. The breakdown of organic matter in the wastewater to carbon dioxide and water is an aerobic process, so air must be provided. Aeration alone uses 50–70% of the total energy consumption in wastewater treatment plants in China.^27^ Given that aeration of wastewater is the major energy user in Chinese wastewater treatment plants, focusing on energy efficiency in this area through optimisation of the activated sludge process is important. Another alternative is to shift the focus from increasing efficiency of aeration to increasing the quantity of organic matter that is retrieved during primary treatment.^30^

SBR and oxidation ditch are variations on the conventional activated sludge process. These processes are popular in China, particularly in plants sized below 20 × 10^4^ m^3^/day, because they are simple to build and easy to manage.^26^, ^29^ In the case of large (20–50 × 10^4^ m^3^ /day) or very large (>50 × 10^4^ m^3^/day) plants, AAO is a much more popular secondary treatment. This is an activated sludge process, as most organic matter is removed in the presence of air, and sludge is returned from the secondary sedimentation tank to the beginning of the process. This requires energy. Nitrogen removal in the AAO process also requires significant aeration (and thus energy) due to differing growth rates for nitrifying and carbon removal organisms.

### 3.3 Sludge Treatment

In Chinese WWTPs, sludge treatment contributes 4.1–13.9% to the total energy consumption within a wastewater treatment plant.^26^ The main processes used for sludge treatment in China are thickening, conditioning and dewatering.^8^ The aim of thickening, conditioning and dewatering is to reduce the water content of sludge, which can drastically reduce the space occupied by sludge and the cost of disposal, as well as making it easier to reuse or recover energy from. Gravity thickening is the most common thickening method. These types of thickeners require electricity to run sludge scrapers that rotate on the bottom of the tank but use less electricity than other thickening methods like centrifuge thickening, which uses centrifugal force to increase the rate at which particles settle.^31^ For example, gravity thickening uses 0.0019–0.0021 kWh m^–3^ for plants servicing over 50 000 people, compared to 0.015–0.035 kWh m^–3^ for thickening centrifuge and floating.^31^ Sludge thickening used 0.4% of total energy consumed at Beijing's Gaobeidian WWTP.^28^

The main dewatering processes used in China are belt, centrifugal and plate‐frame dewatering.^8^ These processes all require energy to provide pressure or centripetal force and this process tends to be more energy consuming than the thickening stage of the sludge treatment process. Dewatering by plate and frame filter press has similar electricity requirements (100 kWh per dry ton of sludge) to centrifugal dewatering (108 kWh per dry ton) but tends to be more effective at reducing the water content of sludge (can achieve 60% water content compared to 80% for centrifugal).^32^ The dewatering process accounted for 1% of energy use at Beijing's Gaobedian WWTP.^28^

**Figure 3:**
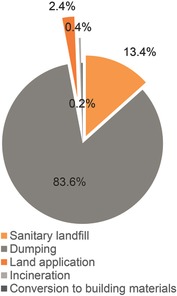
Main disposal methods of sludge in China.^8^

After treatment, a large amount of sludge in China is sent to sanitary landfills (13.4%) or dumped (83.6%). with only a small percentage applied to land (2.4%), incinerated (0.36%) or used in building materials (0.24%) (see **Figure** **3**).^8^ The choice of disposal is linked to cost and regulations regarding water content.^8^ Landfilling is by far the cheapest legal method of sludge disposal but sanitary landfills allow a maximum of 60% water content, which is why many companies choose the even cheaper method of illegal dumping.^8^ Landfilling is also the least electricity‐intensive sludge disposal method, along with brick manufacturing, with both requiring 70 kWh/ton.^32^ Disposal as fertilizer requires 120 kWh/ton, incineration requires 200 kWh/ton and cement manufacturing using sludge is the most energy intensive (250 kWh/ton) because of the high temperature required.^32^ Landfilling may use the least electricity but mono‐incineration of sludge produces the fewest greenhouse gases overall if energy is recovered in the process.^32^ Use of anaerobic digestion for sludge treatment and energy recovery is uncommon in China. Of around 3500 WWTPs in 2013, fewer than 50 had anaerobic sludge digestion processes in China.^8^ The major method of energy recovery used in wastewater treatment in other countries (i.e. combustion of biogas from anaerobic sludge digestion) is relatively uncommon in China. Thus, there is considerable potential to increase energy recovery and reduce overall energy for water and wastewater by expanding use of AD (e.g. a Chinese plant of capacity 600 000 m^3^/day and influent COD of 400 mg/L could recover 0.2 kWh per cubic metre of water from organic energy).^33^

## 4 Conclusion

This article reviewed the current status of energy for conventional water supply and wastewater treatment for urban areas of China. Electricity use by the water and wastewater industry in China has increased significantly over the last decade and a half. Much of the electricity is used to supply locally sourced groundwater and surface water and for primary and secondary wastewater treatment and sludge disposal. Electricity for conventional water supply and wastewater treatment currently accounts for less than 1% of China's total electricity use.

Most energy used by water companies tends to be for sourcing water and distributing it to customers. Standard treatment for water is often very similar (e.g. coagulation, sedimentation, filtration and disinfection for locally sourced surface water) and does not require much energy. Central distribution of water is a major energy user in both Beijing, northern China – where groundwater is the main source – and Changzhou, southern China, where water supplied is mainly surface water. Pumping of water within buildings is another significant energy user that tends to be overlooked.

Biological treatment is the main energy user within wastewater treatment plants in China, with aeration being the major energy burden. The scale of wastewater treatment can affect energy use and the most energy efficient scale depends on the type of secondary treatment technology used. Energy use for sludge treatment and disposal may increase significantly in the future for two reasons. Firstly, wastewater treatment capacity is increasing rapidly, leading to large increases in the quantity of sludge produced. Secondly, most of the sludge produced in China is currently disposed of by inappropriate dumping. Increasing the percentage that is disposed of in legal landfills means meeting the maximum water content requirement of landfills and may lead to an increase in sludge treatment energy.

## Conflict of Interest

The authors delcare no conflict of interest.
